# A rare case of PSA-negative metastasized prostate cancer to the stomach with serum CEA and CA19-9 elevation: a case report

**DOI:** 10.1186/s40792-020-01074-7

**Published:** 2020-12-02

**Authors:** Koji Shindo, Kenoki Ohuchida, Taiki Moriyama, Fumio Kinoshita, Yutaka Koga, Yoshinao Oda, Masatoshi Eto, Masafumi Nakamura

**Affiliations:** 1 Department of Surgery and Oncology, Graduate School of Medical Sciences, Kyushu University, 3-1-1 Maidashi, Higashi-ku, Fukuoka, 812-8582 Japan; 2 Department of Urology, Kyushu University Hospital, Fukuoka, Japan; 3 Department of Anatomic Pathology, Pathological Sciences, Graduate School of Medical Sciences, Kyushu University, Fukuoka, Japan

**Keywords:** Metastasized gastric cancer, Prostate cancer, CEA, CA19-9, PSA

## Abstract

**Background:**

Metastatic cancer to the stomach is relatively rare. Prostate-specific antigen (PSA) is a reliable biomarker used in the screening and management of patients with prostate cancer. However, it is difficult to definitively diagnose a PSA-negative metastatic gastric tumor of prostate cancer because the cancer sometimes resembles primary gastric cancer in clinical images. It is also difficult to distinguish metastatic cancer from primary cancer even in the pathological examination of biopsy samples when the lesion is poorly differentiated adenocarcinoma. There is a possibility that the characteristics of the cancer are changed during treatment such as chemotherapy or radiation therapy. Therefore, careful consideration is required for surgical indication.

**Case presentation:**

A 60-year-old male underwent radical prostatectomy and subsequent radiation therapy for advanced prostate cancer (pT3N1M0) 10 years previously, and hormone therapy was started for metachronous multiple bone metastasis 10 months before. Upper gastrointestinal endoscopy revealed an irregular depressed lesion with a convergence of folds at the greater curvature of the upper gastric body. Biopsy showed poorly differentiated adenocarcinoma that was negative for PSA upon immunohistochemistry. He had high serum carcinoembryonic antigen (CEA) (946.1 ng/ml) and carbohydrate antigen 19-9 (CA19-9) (465.1 U/ml) levels with no elevation of PSA (0.152 ng/ml). The tumor was diagnosed as primary gastric cancer based on the clinical imaging and pathological examination of the biopsy sample including the PSA staining. Based on the diagnosis, laparoscopic proximal gastrectomy with lymphadenectomy was performed. However, pathological examination of the resected specimen revealed poorly differentiated adenocarcinoma that was positive for other prostate markers such as androgen receptor. Thus, the patient was diagnosed with metastasized prostate cancer to the stomach.

**Conclusions:**

We report a case of metastatic gastric cancer of prostate cancer 10 years after radical prostatectomy. In the present case, it was difficult to diagnose a metastatic gastric tumor of prostate cancer preoperatively, because of its resemblance to primary gastric cancer without PSA expression and no serum PSA elevation. Although a rare case entity, it is important to consider the possibility of a metastatic gastric tumor when the surgical indication is determined in cases with another co-existing cancer.

## Background

Metastatic cancer to the stomach is relatively rare, and an incidence rate of 5.4% was reported among 6380 autopsy cases [1]. Of these, malignant melanoma was the most frequent, followed by breast, esophagus, and lung cancer; only two cases (2.5%) of prostate cancer metastasized to the stomach were identified [1]. Among other cancers, bone and the lung are the most common distant metastatic sites in prostate cancer, which is rarely observed in the stomach/bowel (1.8%) [2]. Thirteen cases of metastatic prostate cancer to the stomach were reported in the English literature to date [3–14]. Most were diagnosed by biopsy with immunohistochemistry (IHC) of prostate-specific antigen (PSA), but only one case diagnosed as primary gastric cancer because of negative PSA staining was resected endoscopically [12]. The reported case had prostate cancer with bone metastasis as same as our case, but his serum prostate-specific antigen (PSA) level was high (7040 ng/ml) before treatment [12], which is inconsistent with the finding in our case.

The clinical characteristics and appearance of metastatic tumors in the stomach were previously described [1, 8, 15]. Regarding endoscopic diagnosis, there are two main patterns: one resembling a submucosal tumor, and the other resembling primary gastric cancer [15]. Therefore it is difficult to diagnose the metastatic cancer definitively by endoscopy. Also, it is difficult to diagnose metastatic cancer based on the findings of hematoxylin–eosin (HE) staining of biopsy samples when that cancer was poorly differentiated adenocarcinoma.

PSA is a reliable biomarker of prostate cancer used in the screening and management of patients with prostate cancer [16]. However, in patients with androgen-independent prostate cancer, half (69/141 cases, 48.9%) had elevated carcinoembryonic antigen (CEA) with no correlation with PSA level [17]. IHC of PSA is usually performed to achieve a diagnosis of prostate cancer [18, 19], but there is a possibility that the characteristics of the cancer are changed during treatment with chemotherapy or radiation therapy [19, 20].

Herein, we report a case of PSA-negative metastatic gastric tumor of prostate cancer with serum CEA and carbohydrate antigen 19–9 (CA19-9) elevation 10 years after radical prostatectomy and radiation therapy.

## Case presentation

A 60-year-old male underwent radical prostatectomy and subsequent radiation therapy for advanced prostate cancer (pT3N1M0) 10 years prior, and hormone therapy was initiated for metachronous multiple bone metastasis 10 months before. Upper gastrointestinal endoscopy examination revealed early gastric cancer that did not meet the criteria for endoscopic resection because of the size, depth, and histological type. He was then referred to our department for surgical management. His prostate cancer required continual chemotherapy, but his prognosis depended on the gastric tumor when his chemotherapy showed good effectiveness. The results of ALSYMPCA Clinical Trials [21] revealed that median overall survival was 14.9 months when radium-233 was used in patient with multiple bone metastases without visceral metastases of prostate cancer. His medical history was hypertension, hyperlipidemia and diabetes mellitus, which was treated by medication. He also underwent radiation therapy for left glottic cancer 2 years before.

**Fig. 1:**
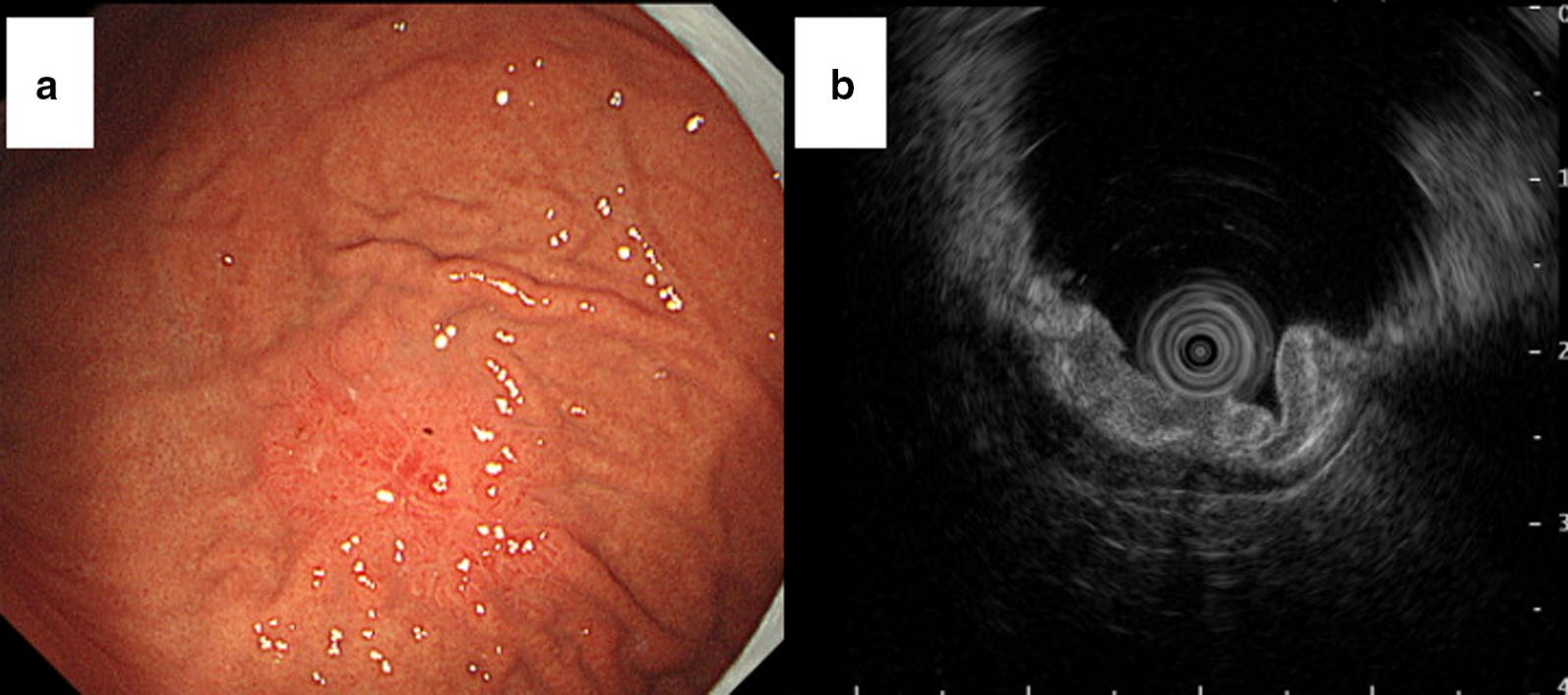
Endoscopic findings. **a** An irregular depressed lesion with convergence of folds of 25 mm in size at the greater curvature of the upper gastric body. **b** EUS showed that the tumor was located mainly at the second layer with partial compression of the third layer

**Fig. 2:**
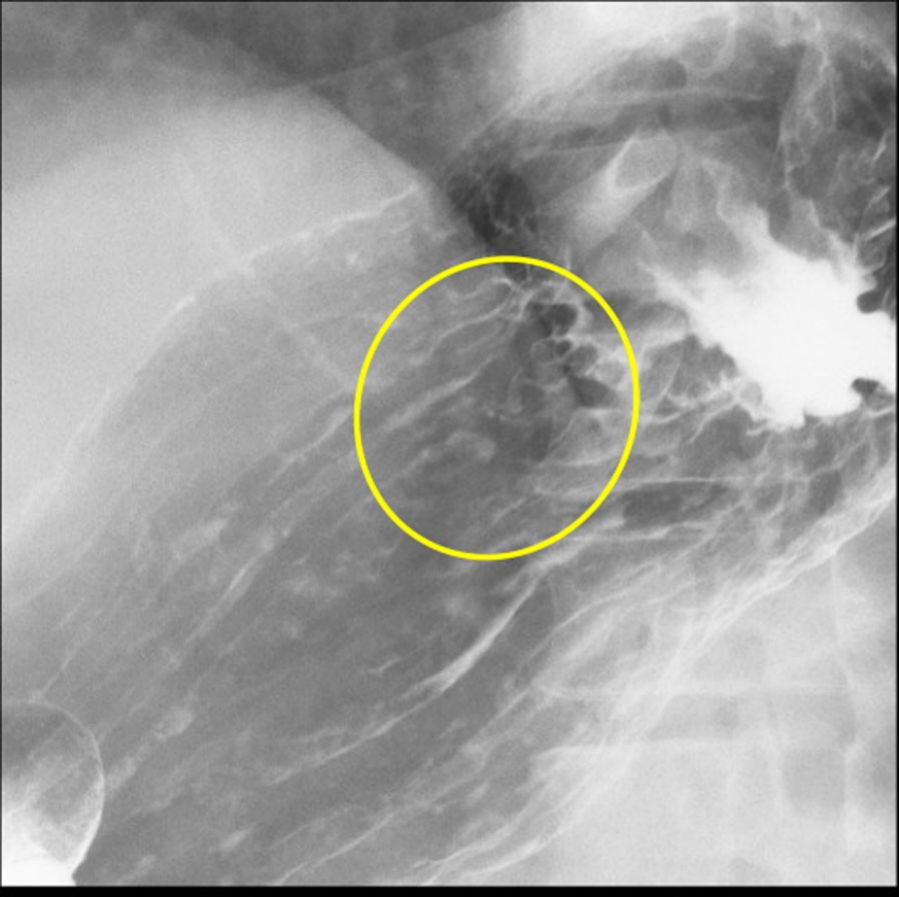
Upper gastrointestinal series showing that the tumor had a convergence of folds suggesting submucosal invasion

**Fig. 3:**
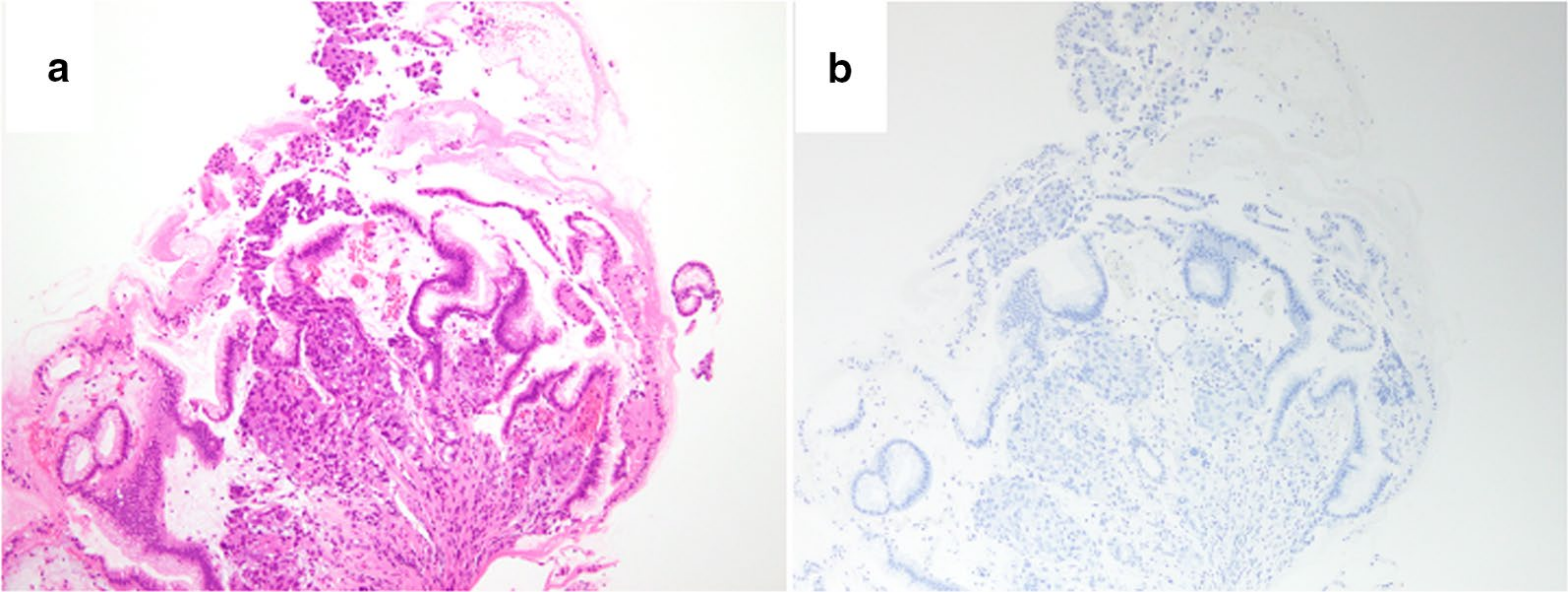
Biopsy specimen.** a** Hematoxylin–eosin staining showing poorly differentiated adenocarcinoma. **b** There was no PSA expression upon IHC. **a** and **b** original magnification × 100

**Fig. 4:**
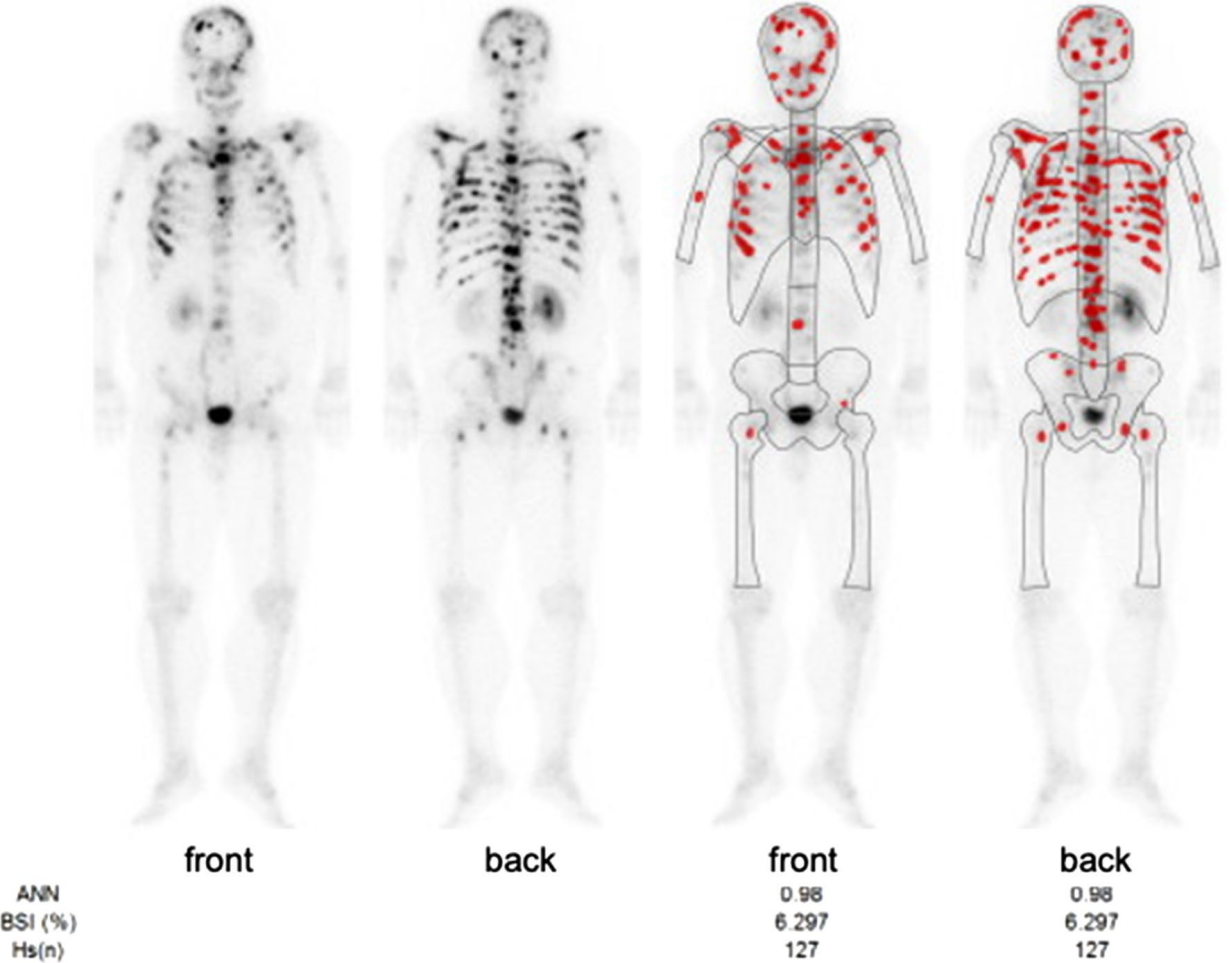
Bone scintigraphy revealed multiple bone metastases resulting from prostate cancer

Physical examination revealed no abnormal finding except for the prostatectomy incision. Laboratory data revealed elevated CEA (946.1 ng/ml) and CA19-9 (465.1 U/ml). His PSA was elevated to 16.84 ng/ml at the time of prostatectomy, but was decreased to 0.193 ng/ml after surgery and radiation therapy. PSA was slightly elevated (4.36 ng/ml) when his multiple bone metastases were identified, but it decreased smoothly following hormone therapy (0.152 ng/ml). On endoscopy, there was a convergence of folds with club-like thickening 25 mm in size at the upper gastric body (Fig. 1a). Endoscopic ultrasound (EUS) examination identified that this tumor was located mainly at the second layer with a partial compression of the third layer, which suggested early gastric cancer invading to the submucosa (Fig. 1b). The cancer was diagnosed as type 0–IIc early primary gastric cancer. Upper gastrointestinal series showed that tumor had a convergence of folds suggesting submucosal invasion. There was no definite wall deformity, but submucosal invasion was indicated by the thickness of the folds (Fig. 2). Biopsy and IHC revealed poorly differentiated adenocarcinoma (Fig. 3a) without PSA expression (Fig. 3b), which suggested the cancer was a primary gastric cancer. The tumor and enlarged regional lymph nodes around the stomach were not recognized on computed tomography (CT), but multiple bone metastases were seen because of prostate cancer on bone scintigraphy (Fig. 4).

**Fig. 5:**
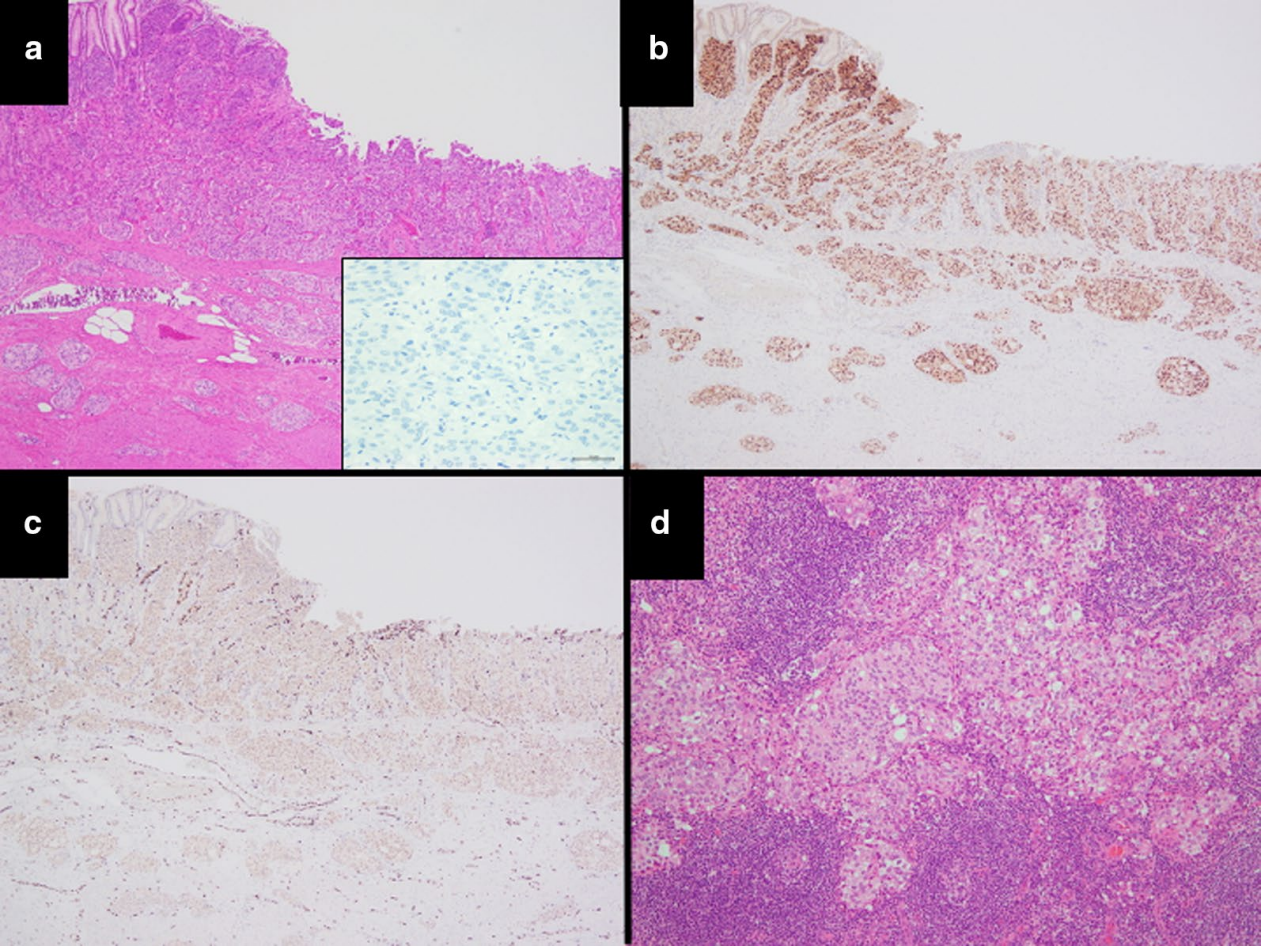
Histopathological findings.** a** There was a proliferation of tumor cells with round-to-oval nuclei and eosinophilic to clear cytoplasm arranged in small nests or a cribriform-like pattern in the gastric wall. The tumor cells were negative for PSA (**a**, inset), but positive for **b** androgen receptor, and **c** ERG in IHC. **d** Lymph nodes were metastasized by the cancer cells around the stomach. **a**, **b, c** × 40, **d** × 100, a inset × 400 original magnification

Following a clinical diagnosis of early gastric cancer with submucosal invasion without metastasis, laparoscopic proximal gastrectomy with D1 + lymphadenectomy was performed. The operating time was 412 min, and the blood loss volume was 195 g. Post-operative pathological examination revealed poorly differentiated adenocarcinoma (Fig. 5a). The tumor was 60–45 mm in size, and mucin production was highlighted by periodic acid Schiff (PAS) and diastase-digested PAS staining. In addition, the tumor cells were negative for PSA (Fig. 5a, inset), but positive for androgen receptor (Fig. 5b), v-ets erythroblastosis virus E26 oncogene homolog (ERG) (Fig. 5c), and alpha-methylacyl-CoA racemase (AMACR). Lymphovascular invasion was frequently seen. Five regional lymph nodes around the stomach were also metastasized by carcinoma cells (Fig. 5d): n#1 (2/12), n#2 (1/5), n#3a (0/2), n#4sa (0/0), n#7 (0/6), n#8a (1/9), n#9 (0/7), and n#11p (1/5). Taken together, these findings suggested the tumor was a metastasized prostate cancer to the stomach with regional lymph nodes metastasis. Postoperatively, the patient followed an uneventful course with no complications, and was discharged at 14 post-operative days. His CEA (849.8 ng/ml) and CA19-9 (538.0 U/ml) did not decrease after gastrectomy. He has continued to undergo chemotherapy (docetaxel) for prostate cancer for 8 months.

## Discussion

Metastatic gastric tumor of prostate cancer is rare and only 13 cases have been reported to date [2–14]. Time to metastasis to the stomach from a diagnosis of primary prostate cancer varies (identified simultaneously to 10 years later). All the available previous cases had serum PSA elevation at the time of metastasis to the stomach. In the present case, the patient underwent radical prostatectomy with radiation therapy to the pelvic cavity previously, and gastric metastasis was detected 10 months after the diagnosis of the bone metastasis. Serum PSA level changed according to the progression of prostate cancer and treatment effect. This case had no elevation of PSA when the gastric metastasis was found, but serum CEA and CA19-9 levels were extraordinarily high, suggesting that these serum markers are not definitive for differentiating a metastatic gastric tumor of prostate cancer from primary gastric cancer.

Elevated tumor markers such as CEA and CA19-9 are common in hepatobiliary cancer and gastrointestinal cancer, but not in prostate cancer. Such unusual elevation of these markers in cancer sometimes indicates poor differentiation. Guthman et al. reported that there are two types of primary prostate adenocarcinoma with selective metastatic spreading [22]. These two types differed in their expression pattern of CEA and PSA. PSA-positive/CEA-negative cancer cells were metastasized to lymph nodes, but CEA-positive/PSA-negative cancer cells were found in liver metastasis [22]. In this case, the characteristics of the cancer cells were similar to the latter, but there was no liver metastasis. Surprisingly, metastasized prostate cancer cells were detected in the regional lymph nodes of the stomach. This is the first report showing regional lymph node metastasis around the metastatic site of prostate cancer.

In the present case, previous pathological examination revealed that the primary prostate cancer was well to moderately differentiated adenocarcinoma accompanied by lymph node metastasis. However, the metastatic lesion to the stomach showed poor differentiation without PSA expression in the biopsy sample. Based on the clinical imaging and the pathological examination, the present tumor was diagnosed as primary gastric cancer preoperatively. Then, we performed gastrectomy with lymph node dissection. However, the IHC analyses of the resected tumor revealed that tumor was positive for other markers of prostate cancer, such as AMACR, prostate-specific acid phosphatase (PSAP), androgen receptor, and ERG, suggesting that the tumor was not primary gastric cancer but a metastatic lesion of prostate cancer. There was only one previous case of metastatic gastric metastasis of prostate cancer, which was negative for PSA, similar to our case [12], suggesting that PSA staining was not enough to differentiate the prostate cancer from other cancers. Also, these cases suggest that it is important to perform IHC of the preoperative biopsy sample with multiple markers when the metastatic cancer cannot be denied based on the medical history.

In the present case, there was a difference in the differentiation between the primary lesion of prostate cancer and the biopsy sample derived from the stomach lesion. This difference is one of the main reasons why we undertook gastrectomy when the surgical indication was discussed preoperatively. Previously, it was reported that radiation therapy for primary prostate cancer decreased the number of poorly formed glands and induced nuclear pyknosis resembling poor differentiation [19, 20]. In the present case, radiation therapy might also have affected remnant prostate cancer cells after prostatectomy in terms of the differentiation, leading to the difficulties in preoperative diagnosis based on the pathological examination of the biopsy sample.

The median overall survival of patients with bone and visceral metastases of prostate cancer is only 14 months [23]. In multivariate analysis, visceral metastasis was an independent prognostic factor when the patients had lymph node metastasis of prostate cancer [23]. Previously, it was reported that metastatic prostate cancer with CEA elevation had poor prognosis with aggressive behavior [17, 24]. In the present case with visceral metastasis and CEA elevation, careful follow-up will be needed although docetaxel therapy has continued for 8 months after gastrectomy without disease progression.

## Conclusion

In the present case, a metastatic gastric tumor of prostate cancer 10 years after radical prostatectomy was surgically resected. The pathological examination showed gastric regional lymph node metastasis with elevated CEA and CA19-9 without PSA elevation. It was difficult to diagnose the metastatic gastric tumor preoperatively in the present case because of the findings resembling primary gastric cancer on the preoperative imaging examination and the pathological findings of poor differentiation without PSA expression in preoperative biopsy samples. Although a rare case entity, it is important to consider the possibility of a metastatic gastric tumor with no PSA expression when the surgical indication is determined for gastric tumors in patients with a risk of prostate cancer recurrence.

## Data Availability

Not applicable.

## Abbreviations

IHC: Immunohistochemistry
PSA: Prostate-specific antigen
CEA: Carcinoembryonic antigen
CA19-9: Carbohydrate antigen 19-9
CT: Computed tomography
EUS: Endoscopic ultrasound
AMACR: Alpha-methylacyl-CoA racemase
PSAP: Prostate-specific acid phosphatase
ERG: V-ets erythroblastosis virus E26 oncogene homolog
